# Cell-to-Cell Stochastic Variation in Gene Expression Is a Complex Genetic Trait

**DOI:** 10.1371/journal.pgen.1000049

**Published:** 2008-04-11

**Authors:** Juliet Ansel, Hélène Bottin, Camilo Rodriguez-Beltran, Christelle Damon, Muniyandi Nagarajan, Steffen Fehrmann, Jean François, Gaël Yvert

**Affiliations:** 1 Université de Lyon, Lyon, France; 2 Laboratoire de Biologie Moléculaire de la Cellule, Ecole Normale Supérieure de Lyon, CNRS, Lyon, France; 3 IFR128 BioSciences Lyon-Gerland, Lyon, France; 4 Laboratoire de Biotechnologie et Bioprocédés, Institut National des Sciences Appliquées, Toulouse, France; University of Oxford, United Kingdom

## Abstract

The genetic control of common traits is rarely deterministic, with many genes contributing only to the chance of developing a given phenotype. This incomplete penetrance is poorly understood and is usually attributed to interactions between genes or interactions between genes and environmental conditions. Because many traits such as cancer can emerge from rare events happening in one or very few cells, we speculate an alternative and complementary possibility where some genotypes could facilitate these events by increasing stochastic cell-to-cell variations (or ‘noise’). As a very first step towards investigating this possibility, we studied how natural genetic variation influences the level of noise in the expression of a single gene using the yeast *S. cerevisiae* as a model system. Reproducible differences in noise were observed between divergent genetic backgrounds. We found that noise was highly heritable and placed under a complex genetic control. Scanning the genome, we mapped three Quantitative Trait Loci (QTL) of noise, one locus being explained by an increase in noise when transcriptional elongation was impaired. Our results suggest that the level of stochasticity in particular molecular regulations may differ between multicellular individuals depending on their genotypic background. The complex genetic architecture of noise buffering couples genetic to non-genetic robustness and provides a molecular basis to the probabilistic nature of complex traits.

## Author Summary

Although most inter-individual phenotypic variabilities are largely attributable to DNA differences, a wealth of examples illustrate how a single biological system can vary stochastically over time and between individuals. Identical twins are not identical, and similarly, clonal microbial cells differ in many aspects even when grown simultaneously in a common environment. Using yeast as a model system, we show that a population of isogenic cells all carrying genotype A showed higher cell-to-cell heterogeneity in gene expression than a population of isogenic cells of genotype B. We considered this level of intra-clonal heterogeneity as a quantitative trait and performed genetic linkage (on AxB) to search for regulators of it. This led to the demonstration that transcriptional elongation impairment increases stochastic variation in gene expression *in vivo*. Our results show that the two levels of inter-individual diversity, genetic and stochastic, are connected by a complex control of the former on the latter. We invite the community to revisit the interpretation of *incomplete penetrance*, which defines cases where a mutation does not cause the associated phenotype in all its carriers. We propose that, in the case of cancer or other diseases triggered by single cells, such mutations might increase stochastic molecular fluctuations and thereby the fraction of deviant cellular phenotypes in a human body.

## Introduction

Two fascinating area of research on gene expression have been conducted intensively and independently during the past couple of years. A large community of geneticists has contributed to the identification of genetic sources underlying expression differences between individuals. Such expression Quantitative Trait Loci (eQTL) were first mapped in maize[1], yeast[2] and mouse[3] and consecutively identified in many organisms including worms[4], *A. thaliana* [5] and humans[6],[7]. All these studies shared three important conclusions: gene expression levels differ greatly among individuals of a species, their genetic control is complex, and despite the high number of statistical tests required, genetic mapping of regulators is feasible on a genome×transcriptome scale. In addition, promising methods have emerged to extract causal relationships among molecular regulations[8]–[10], illustrating how expression data can power genetic linkage or association studies. Recently, the genetics of gene expression appeared even more complex when discovering the high degree of variation in human transcript isoforms [11]. This complexity of molecular regulations, which very likely underlies the genetics of complex traits, is now anticipated and integrated in many designs. However, like the large majority of molecular regulations described to date, these observations were made on samples of many (10^4^–10^9^) cells and therefore reflect only averages of cellular states. This limitation can be very frustrating when studying traits such as cancer that can emerge from a single or very few cells.

Simultaneously, another large community of scientists from various disciplines has been investigating the sources and properties of stochastic fluctuations in gene expression. These investigations were powered by the development of single-cell reporter assays. Following previous terminology, we will refer here to *noise* in gene expression as the stochastic variation of a protein concentration among isogenic cells, grown homogeneously in a common environment. This noise was demonstrated to contribute to non-genetic cellular individuality[12]–[16]. Although non-deterministic fluctuations in gene expression can be detrimental to cellular physiology, they can also provide a mechanism of single-cell memory[17]–[19] and shape differentiation during development[20]. Notably, high noise was observed in old mice hearts suggesting that age-related health decline could result from such stochastic fluctuations[21]. Genetic sources of noise in gene expression were also investigated. So far, the list of factors shown experimentally to contribute to noise includes the SWI/SNF, INO80 and SAGA chromatin modification complexes[22], TATA-box mutations[22],[23], MAP Kinases implicated in the response to yeast pheromones[24], the Swi4 transcriptional activator[25], DNA topology[13] and ribosomal activity in bacteria[26]. This list will very likely increase dramatically in the near future as investigations of single-cell expression levels are becoming more and more popular. In addition, the topology of gene regulatory networks has clearly been shown to drive various levels of instabilities, for example via the presence/absence of functional feedback loops[17].

We present here a study bridging these two fields of investigations, by considering noise in gene expression as a quantitative trait. We quantified noise of a representative reporter system in various strains of *S. cerevisiae* and found reproducible differences among strains. Genetic segregation of noise values revealed a complex genetic control, and Quantitative Trait Loci mapping allowed the identification of three loci modulating noise levels. One locus led to the identification of transcriptional elongation as an additional source of noise. Based on these observations from a yeast model, we propose a new interpretation of the incomplete penetrance observed for common traits that are triggered by single cells in higher eukaryotes.

## Results

### Natural Genetic Variation of Noise in Gene Expression

**Figure 1:**
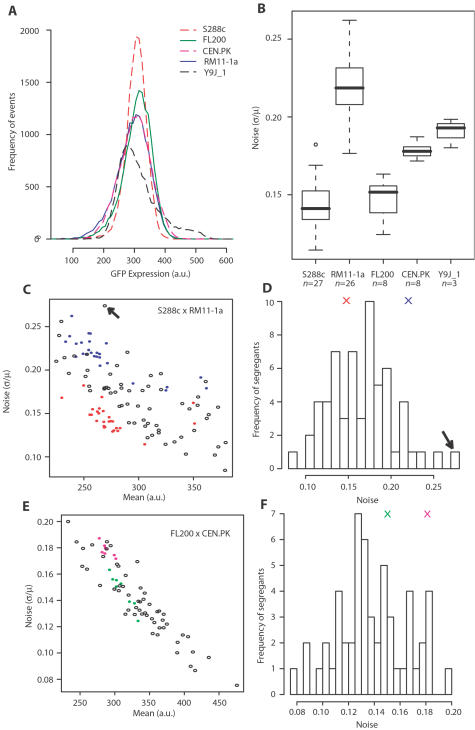
Strain-to-strain variation and complex genetic segregation of noise. A) Five representative flow-cytometry experiments on strains GY51, GY43, GY44, GY53 and GY445 derived from S288c, FL200, CEN.PK, RM11-1a and Y9J_1 respectively, each showing the distribution of P_MET17_-GFP expression levels in 15,000 individual cells (events) after two hours of moderate induction. Raw fluorescent values were corrected for cell size and granularity as described in Materials and Methods. Mean expression levels were similar between strains, while variances differed. B) Boxplot representation of flow-cytometry experiments repeated *n* times in the same conditions as in A), showing reproducible noise differences between genetic backgrounds. C–D) Genetic segregation of P_MET17_-GFP noise in a cross between S288c and RM11-1a backgrounds. Colored dots in C) represent independent flow-cytometry experiments performed on strain GY51 (red) or strain GY53 (blue). Each open circle represents the average values of three experiments performed on one S288c×RM11-1a segregant. The distribution of noise values in these segregants is shown in D), with the average noise of GY51 and GY53 represented as red and blue crosses, respectively. The arrow points to segregant GY157 displaying extremely high noise. E–F) Genetic segregation of P_MET17_-GFP noise in a cross between FL200 and CEN.PK backgrounds. Representation is similar as in C) and D), with repeated experiments on strain GY43 and GY44 shown in green and magenta, respectively. One flow-cytometry experiment was performed on each segregant obtained by crossing GY43 and GY44 (open circles). All segregants analyzed possessed the *ura3-52* mutation of GY44, and their differences must therefore result from allelic variations residing in other genes.

To investigate the natural genetic diversity of noise in the expression of a representative gene, we integrated in the genome of five distant *S. cerevisiae* strains a reporter construct where the green fluorescent protein (GFP) was regulated by the inducible promoter of the *MET17* (*YLR303W*) gene. The strains used were three unrelated laboratory strains (S288c, FL200 and CEN.PK), a wine strain from California (RM11-1a), and a wine strain from Japan (Y9J_1). In each case the construct was integrated at the same *HIS3* chromosomal locus. We then quantified the level of expression in individual living cells by flow cytometry. Figure 1A shows representative experiments where 15,000 cells were recorded for each background after two hours of moderate induction. We found that although mean induction was similar between backgrounds, the variance of gene expression level differed. This observation was reproduced when the experiments were repeated at various dates (Figure 1B). This suggested the presence of genetic variation that might control noise without necessarily affecting mean expression of the cell population. To see if the difference in noise between S288c and RM11-1a was specific to the chromosomal environment of the *HIS3* locus, we integrated the same reporter system at the *LYS2* locus located on another chromosome (Figure S1). Noise and mean expression values were comparable to the results obtained when targeting *HIS3*, showing that the difference in noise between the two strains could not be accounted for by differences at the integration locus only.

### Noise as a Complex Trait

If strain-to-strain difference in noise levels is under genetic control, it should be heritable. To determine if this was the case, we integrated the *P_MET17_-GFP* construct at the *HIS3* genomic locus of 61 segregants issued from a cross between S288c and RM11-1a, two backgrounds displaying different noise levels. Noise was estimated from triplicate experiments for each segregant. This showed that noise segregated as a quantitative phenotype, with evidence of a polygenic control (Figure 1C–D). Heritability was high (81%) and the continuous, Gaussian-like distribution of noise values among segregants excluded simple Mendelian inheritance. In addition, a few segregants showed noise values outside the range of parental values (transgression), suggesting segregation of alleles with opposite effects. Importantly, mean expression (the average fluorescence of the population of cells) also segregated continuously, and the two traits (noise and mean) were correlated (R^2^ = 0.51, *P* = 5×10^−11^ from linear regression). This scaling between mean expression and noise level is consistent with previous observations[14],[27],[28]. In the case of our genetic design, this scaling of segregant values indicate the presence of genetic loci acting on both mean and noise, although mean values did not differ between the parental backgrounds. This apparent discrepancy can be explained by alleles with opposite effects that compensate mean expression in the parental strains (higher transgression for mean than for noise).

To examine further the natural genetic segregation of noise, we analyzed a cross from another pair of unrelated backgrounds. We crossed GY43 with GY44, two strains carrying the *HIS3:P_MET17_-GFP* insertion and derived from FL200 and CEN.PK, respectively. Random spores were generated and were considered further only if they were auxotroph to uracil, to avoid the presence of diploid contaminants. Noise was measured in 55 of these spores, and the distribution obtained also showed high heritability (88%) with a continuous genetic segregation and evidence of transgression (Figure 1E–F). In addition, noise values of GY43xGY44 segregants were enriched for low levels and were not centered at the mid-parental value. This is probably not a bias from our selective choice of *ura3* segregants because average noise was also globally low among spores of dissected tetrads (Figure S2). This asymmetry towards low noise is more likely due to the presence of interacting alleles, a particular combination of which being required to confer high noise (epistasis).

### Quantitative Trait Locus Mapping of Noise

**Figure 2:**
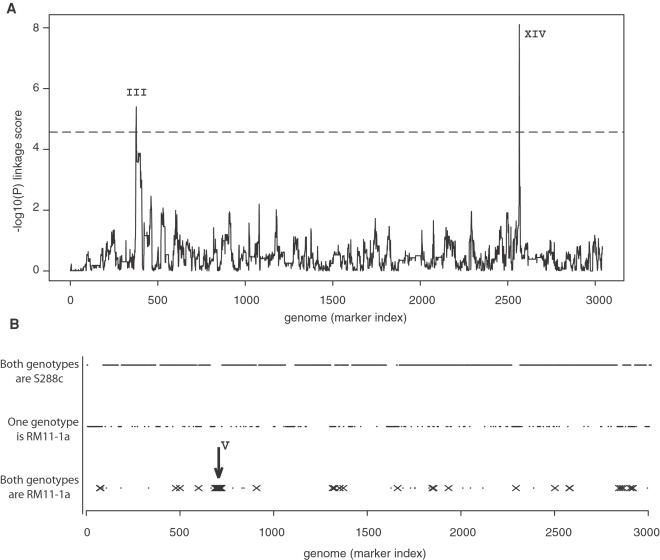
Genome scans for noise QTL. A) Noise levels of P_MET17_-GFP from S288c×RM11-1a segregants were treated as a quantitative phenotype and genetic linkage was tested at each of 3042 marker positions on the genome. Markers were ordered by their physical position on the reference genome S288c, from chromosome I to chromosome XVI. At every marker, the y-axis represents the -log_10_(*P*) linkage score, where *P* is the nominal *P*-value of the test. The dashed line indicates the 1% genome-wide significance threshold. Two significant signals (QTL_1_ and QTL_2_) were found on chromosome III and XIV, respectively. B) Cumulative genotypes of two introgressed strains. Haploid strains GY159 and GY174 were constructed by introgressing high P_MET17_-GFP noise level from RM11-1a into S288c. These strains were genotyped at 3015 marker positions using oligonucleotide microarrays. Markers were ordered along the x-axis as in a) and are shown as small dots. The GY159 and GY174 genotypes are presented on three levels depending on whether both strains (bottom), one of them (middle), or no strain (top) inherited the RM11-1a allele of the marker. Among the bottom genotypes, markers that where also inherited from RM11-1a in strain GY157 (the S288c×RM11-1a segregant with highest noise) are marked by crosses. These positions are candidates to contain RM11-1a alleles conferring high noise. A cluster of such candidate markers was found on chromosome V (arrow).

**Figure 3:**
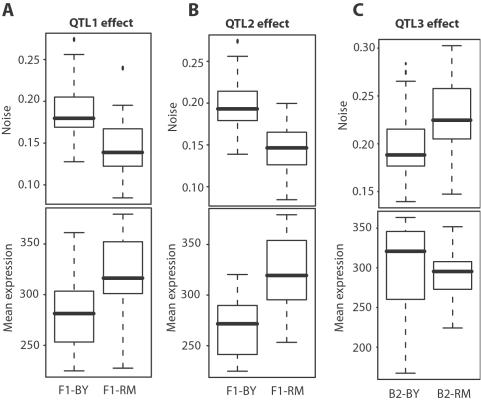
Contributions of QTL to P_MET17_-GFP noise (top panels) and mean expression (bottom panels). A–B) S288c×RM11-1a segregants were separated in two groups (F1-BY and F1-RM) according to their genotype at QTL_1_ (A) or QTL_2_ (B). For both QTL, the inheritance of the RM11-1a allele was associated to lower noise and higher mean expression. The differences in mean expression between the F1-RM and F1-BY groups were highly significant: *P* = 4×10^−6^ (A) and *P* = 7.8×10^−9^ (B). C) Strain GY157 was crossed with a derivative of S288c, and fifty five segregants were characterized. These segregants were separated in two groups (B2-BY and B2-RM) according to their genotype at the candidate region on chromosome V. The RM11-1a allele conferred a significant increase in noise (*P* = 3.5×10^−3^), therefore validating the region as a third QTL (QTL_3_), while no effect of the genotype was observed on mean expression.

We then sought to map genetic variations underlying noise differences between S288c and RM11-1a, which we did by two methods. Firstly, using the noise values of the 61 segregants from S288cxRM11-1a and their genotypes at 3042 marker positions[29], we screened the genome for Quantitative Trait Loci (QTL). Two QTL were found (position 79091 on chromosome III and position 449639 on chromosome XIV) at a genome-wide significance of 1% (Figure 2A). Secondly, we introgressed the high-noise phenotype of RM11-1a into the S288c background, and searched for alleles that had been conserved from RM11-1a in the resulting strains (see Materials and Methods). This approach identified a region on chromosome V (from position 116530 to 207819) as a candidate region for conferring high-noise level (Figure 2B). To validate or refute this locus as a QTL, we backcrossed GY157, the S288c×RM11-1a segregant showing highest noise, with an S288c derivative. Fifty five random spores from this cross were analyzed by flow cytometry to quantify their level of *HIS3:P_MET17_-GFP* noise. We took advantage of the presence of the *ura3Δ0* auxotrophic marker within the region of interest to genotype the 55 spores by plating them on URA-plates. A significant linkage was found between these genotypes and noise levels (Wilcoxon-Mann Whitney test, *P* = 3.5×10^−3^) (Figure 3C), which validated the locus as a third QTL. The three QTL identified showed the following characteristics: Firstly, in all three cases, the molecular control of noise involves *trans*-regulations (a polymorphism in one gene affecting noise level of another gene) because none of the QTL were located at or near the *HIS3* integration site nor the *MET17* endogenous regulatory region. Secondly, QTL_1_ and QTL_2_ but not QTL_3_ were also in genetic linkage with the mean expression levels of the samples (Figure 3). Consistently, QTL_1_ was already detected as an expression QTL (eQTL) locus controlling MET17 mRNA levels in a previous study where only mean expression was measured[29]. This indicated that regulatory variation could scale noise levels by acting on mean expression, raising the possibility that other eQTL identified by ‘genetical genomics’[30] are likely to influence noise as well. Thirdly, and surprisingly, the effects of QTL_1_ and QTL_2_ were opposite to the effects expected from the parental difference: alleles from the high-noise background RM11-1a conferred low noise (Figure 3A–B). This was consistent with the transgressive segregation visible on Figure 1C and it supported the presence of additional QTL (such as QTL_3_) where RM11-1a alleles conferred high noise. Finally, QTL_3_ effect was extremely low in the panel of S288cxRM11-1a segregants (*P* = 0.4 from linear regression). From these observations, we conclude that the difference in noise between S288c and RM11-1a backgrounds can not be attributed to one or a few loci but rather results from the cumulative effects of numerous QTL, several of which remain to be identified.

### Noise Increase Resulting From Uracil Metabolism Impairment

**Figure 4:**
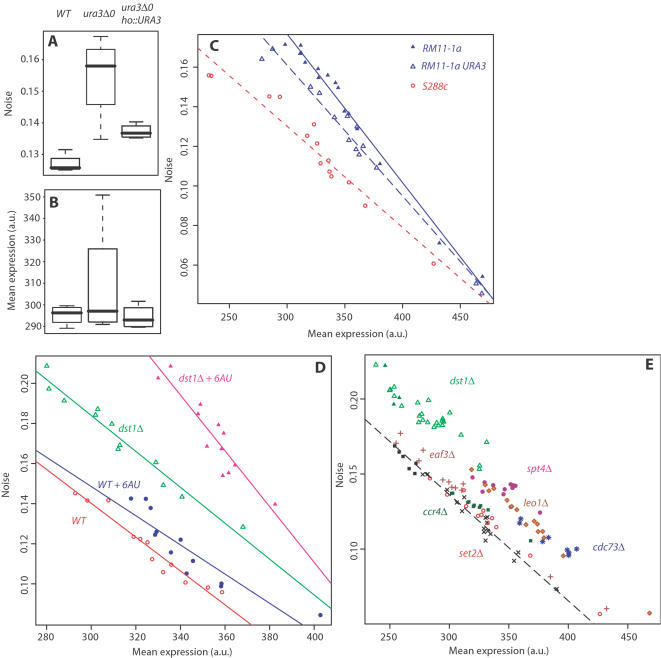
Increased noise resulting from transcriptional elongation impairment. A–B) Comparison of P_MET17_-GFP noise and mean expression levels between S288c-derived strains GY244, GY246 and GY333 that were isogenic except for the specified *ura3* genotypes. C) Complementation of *ura3* in RM11-1a derived strain partially reduced its high-noise phenotype. Strains GY51 (open red circles), GY53 (filled blue triangles) and GY601 (open blue triangles) were compared at various induction strength (Methionine concentration from 0 to 200 µM). Each dot represents one sample of 15,000 cells. Lines indicate linear fits on each strain. D) Additive noise increase in response to 6-azauracil (6AU) and TF_II_S (*dst1*) mutation. Wild-type strain GY51 (circles) and *dst1Δ* strain GY321 (triangles) were cultured with (filled blue, filled magenta) or without (open red, open green) 6AU, at various induction strength as in c). Lines indicate linear fits on each subgroup. E) Comparison of P_MET17_-GFP noise and mean expression levels between various transcription elongation mutants. Strains GY602 (control strain *trp1Δ::KanMX*, black ‘x’), GY321 (*dst1Δ::hisG*, open green triangles), GY358 (*dst1Δ::hisG hoΔ::KanMX*, filled green triangles), GY361 (*dst1Δ::hisG hoΔ::(DST1+KanMX)*, filled black squares), GY603 (*eaf3Δ::KanMX*, brown ‘+’); GY604 (*spt4Δ::KanMX*, purple filled circles), GY605 (*leo1Δ::KanMX*, orange filled diamonds), GY606 (*set2Δ::KanMX*, open red circles), GY607 (*ccr4Δ::KanMX*, filled dark green squares), GY608 (*cdc73Δ::KanMX*, blue stars), that were all isogenic to GY51 except for the specified genotypes were compared at various induction strength. Dashed line represents linear fit to the GY602 control strain data points (no elongation impairment).

The presence of *ura3Δ0* at QTL_3_ prompted us to test if this mutation was responsible for noise modulation. When introduced in the S288c background, a significant increase in *HIS3:P_MET17_-GFP* noise was observed (Figure 4A–B). Consistently, restoring wild-type *URA3* in the resulting mutant or in RM11-1a significantly reduced noise (Figure 4A–C), and another null allele (*ura3-52*) could also increase noise (Figure S3A), as well as treatment with 6-azauracil, a drug inhibitor of the *URA3* gene product (Figure 4D). Since random spores of the FL200×CENPK cross mentioned above displayed low noise despite bearing the *ura3-52* mutation, we examined additional spores from tetrads and found that, as expected, Ura^+^ spores from this cross displayed even lower noise (Figure S2). Surprisingly, increasing the concentration of uracil in the culture medium did not reduce noise of a *ura3Δ0* strain (Figure S3B). This might be due to limiting steps of the import mechanism. Finally, the *ura1Δ* and *ura2Δ* mutations were also found to increase noise levels (Figure S3C). Altogether, these observations validated *ura3* as a responsible gene for QTL_3_ with *ura3Δ0* accounting for most (74%) of the locus effect seen in segregants (Figure 3C and 4A). So if additional noise regulators resided at QTL_3_, we expect their contribution to be minor. The *ura3Δ0* allele is not natural but was introduced in RM11-1a for laboratory purposes unrelated to this study[2]. However, null *ura3* alleles exist in nature: *ura3-52* results from a *Ty* transposable element insertion[31], and when searching the Saccharomyces Genome Resequencing Project[32] we found three additional severe mutations (G->GA, G->GA, and TTG->TAG(stop) at 183, 219 and 94 nucleotides from ATG, respectively) in two unrelated natural isolates (NCYC361 from an Irish brewery and UWOPS87_2421 from a cladode in Hawaii). Also, *ura3* mutations are not the sole source of natural genetic variation in noise, since high noise was found in the Y9J_1 background (a prototrophic strain with functional *URA3*), and since *ura3Δ0* accounted for only 37% of the total noise difference between S288c and RM11-1a (Figure 4C and Materials and Methods).

### Transcriptional Elongation Is Involved in the Control of Noise

Inhibition of uracil synthesis is known to reduce the intracellular pool of nucleotides available for RNA synthesis and this shortage is known to affect transcriptional elongation[33]. To directly test if transcriptional elongation was involved in the control of noise, we measured noise levels in a *dst1Δ* mutant strain lacking TF_II_S activity. A dramatic increase of noise was observed, with no detectable difference in mean expression (Figure 4D). This increase was suppressed when the mutation was complemented by integrating the wild-type *DST1 (YGL043W)* gene at the *HO* locus (Figure 4E). Even higher noise levels were obtained when *dst1Δ* cells were treated with 6-azauracil (Figure 4D), highlighting the gradual noise increase with gradual transcriptional elongation defects. To see which of several known partners of elongating RNA PolII were involved in noise modulation, we measured P_MET17_-GFP noise in strains lacking specific elongation factors (Figure 4E). A pronounced noise increase was observed in *spt4Δ* mutant, and in mutants lacking the Leo1p or Cdc73p subunits of the Paf1 complex. This suggested that recruitment of Paf1 to elongating RNA PolII (a step requiring Spt4p[34]), was involved in noise control. However, full integrity of the Paf1 complex was not essential since noise remained low in the absence of the Ccr4p subunit. Finally, noise remained low in *set2Δ* and *eaf3Δ* mutants, showing that methylation of lysine 36 of histone H3 and recruitment of Rpd3S[35] for histone deacetylation were not involved. Thus, noise appeared to be strongly connected to the facilitation of transcriptional elongation but not to the subsequent resetting of chromatin to an inactive state.

## Discussion

We showed that noise in gene expression can be subjected to natural genetic variation with a complex inheritance pattern in yeast. In agreement with previous studies[27],[28] we observed that natural genetic variation of noise tended to scale with the genetic control of mean expression. However, two divergent backgrounds could differ only in noise while their cross generated segregants varying in both noise and mean. This supports the presence of two classes of alleles: those acting on both traits (such as QTL_1_ and QTL_2_) and those acting specifically on noise (such as *ura3* and *dst1*).

We demonstrated that impairing the progression of transcriptional elongation can increase the level of noise in gene expression. When elongating RNA polymerase II is stalled because of such defects, expression of the corresponding messenger in this particular cell is blocked until transcriptional initiation takes place again. It is therefore not surprising that this stalling increases stochasticity, as compared to a wild-type context where elongation can resume rapidly, and our results are consistent with a previous numerical model of elongation defects[36].

The complex genetic control of noise makes it a potentially evolvable trait. Although our study did not address whether this genetic control correlates with any adaptive mechanism, the results can be discussed in the context of selection. Living systems maintain a delicate balance between robustness and flexibility[37]. The former ensures stability of ‘normal’ physiology, and the latter provides adaptability to environmental changes. Thus, fluctuating environments might maintain flexibility. One consequence of the propagation of many alleles contributing to noise is the production of few individuals in which regulations are highly noisy, the term ‘individual’ here referring to a human being, a yeast strain or a congenic animal or plant breed. The individuals displaying high noise are likely to have reduced fitness in ‘standard’ environments but they may be readily adapted to new environmental conditions. One possible advantage provided by genetic complexity is to generate this ‘reservoir’ of individuals without perturbing the bulk of the population, because most individuals harbour only few of the alleles conferring high noise levels. However, whether evolution in fluctuating environments can shape the genetics of noise control remains to be demonstrated.

Finally we propose to revisit the interpretation of incomplete penetrance for traits that arise from one or very few cells in higher eukaryotes. Despite intense investigations on the genetic predisposition to common traits, it remains unclear why the underlying alleles express their effects in only a fraction of carriers[38]. For example, a fortunate ∼20% of women carrying BRCA2 mutations associated with high-risk of breast cancer do not develop the disease[39]. In default of any clear explanation, this incomplete penetrance is usually interpreted as the result of interactions that remain to be discovered. This assumes that causative genes manifest their effect only if the carrier is exposed to specific environmental conditions (gene×environment interactions) or if the carrier possesses particular alleles at additional genes, yet undiscovered, which unbuffer the effect of the causative gene (gene×gene interactions). This explanation probably holds for many cases of incomplete penetrance, but since the underlying interactions are currently extremely difficult to identify, their involvement generally remains hypothetical.

Many common traits such as cancer, developmental defects, autoimmunity, or infection can result from rare cellular events. Considering the huge number of cells constituting a human body, these traits can emerge from a very slight increase in the probability of such events. It is therefore possible that cases of genetic predisposition to these traits are caused by low-penetrance alleles that simply increase the chances of such events, without driving them deterministically, and therefore increase the frequency of peculiar cells. Under such a scenario, incomplete penetrance would naturally result from the probabilistic nature of the traits, without necessarily requiring complex genetic interactions.

One way to increase, even slightly, the probability of rare cellular events is to increase stochastic fluctuations in their underlying molecular mechanism. Our study showed that in yeast, natural allelic differences can influence the level of noise in a particular molecular regulation. It is likely that similar scenarios are present in higher eukaryotes. An exciting area of investigation would be to re-examine disease-predisposing alleles in terms of their probabilistic effects among single cells of the tissue they target.

## Materials and Methods

### Plasmids

The NatMX cassette was amplified from the integrative plasmid pFvL99 (kindly provided by F. van Leeuwen and D. Gottschling, FHCRC, Seattle) using primers 5′-GCAAGCGATCCGTCCTAAGAAACCATTATTTAAATGGATGGCGGCGTTAGTATC-3′ and 5′-ATCCGCTTACAGACAAGCTGTGACCGTCTCGACATGGAGGCCCAGAATAC-3′ and cloned by gap-repair recombination into pUG23 (a centromeric plasmid carrying yEGFP3[40] under the control of the *MET17* promoter, from J.Hegemann, Düsseldorf, Germany) linearized at BsmBI to generate plasmid pGY6. The ScaI fragment containing replicative and centromeric sequences of pGY6 was replaced by the ScaI fragment of pFvL99 to create pGY8. To generate plasmid pGY12, the *HIS3* gene of pGY6 was replaced by *LYS2* flanking sequences by transforming strain BY4742[41] with pGY6 linearized at NheI with PCR fragment LYS2-UD and recovering the gap-repaired pGY9 resulting plasmid from HIS-NAT^R^ colonies. The LYS2-UD PCR product was obtained by fusing two PCR products, each obtained by amplifying genomic DNA from BY4716[41] with primers 5′-GCATCAGAGCAGATTGTACTGAGAGTGCACCATAAATTCCTAGGAAGCGGTCAGCAAGAAGAAA-3′, 5′-AATATAAGCGGCCGCTCGAGTTTATACAGTACCTTTTTGAACTTCGTC-3′ and primers 5′-TGTATAAACTCGAGCGGCCGCTTATATTCATCATGCTGCGAAGAACTA-3′, 5′-TCCTTACGCATCTGTGCGGTATTTCACACCGCATAGATCCGTCCATGTACAATAATTAAATATGAATTAGG-3′, respectively. The ScaI fragment of pGY9 containing replicative and centromeric sequences was replaced by the ScaI fragment of pFvL99 to create pGY12. For the complementation test of *dst1Δ*, the DST1 gene of strain BY4716 was amplified using primers 5′-GCGAGCTCTCATTTTATCGTTTTCGT-3′ and 5′-CGGAGCTCTTCTTTAGTTCTGACCGA-3′, the product was digested with SacI and cloned into the SacI site of plasmid pHO-poly-KanMX4-HO[42] to give plasmid pHO::DST1.

### Strains

The strains used in this study are listed in Table S1. Plasmid pGY8 was linearized at NheI and integrated at the *HIS3* locus of FL200, CEN.PK113-5D, BY4716 (isogenic to S288c), YEF1685 (a non-clumpy derivative of RM11-1a), Y9J_1 and in 61 F1 segregants from BY4716×RM11-1a described in Brem et al. 2005 to create GY43, GY44, GY51, GY53, GY445 and the set of S288cxRM11-1a *HIS3:P_MET17_-GFP* strains, respectively. At every transformation, cells were separated in three tubes just after heat shock so that recovery in YPD medium and cell division occurred independently before plating each fraction on a separate NAT plate. This way, three independent transformants were obtained each time. Plasmid pGY12 was linearized at XhoI and integrated at the *LYS2* locus of BY4709 and YEF1946 to generate GY122 and GY125 strains, respectively. To introgress the RM11-1a alleles conferring high noise into a global S288c background, GY53 and BY4716 were crossed, a resulting spore with high noise but similar mean was selected and crossed with BY4719, a resulting spore with high noise but similar mean was selected and crossed with FYC20-2A, a resulting spore with high noise but similar mean was selected and crossed with BY4713, and a resulting spore with high noise but similar mean was selected and called GY159. To repeat this procedure in a totally independent way, GY51 and YEF1946 were crossed, a spore with high noise but similar mean was selected and crossed with FY67, a resulting spore with high noise but similar mean was selected and crossed with BY4712, a resulting spore with high noise but similar mean was selected and crossed with BY4715, and a resulting spore with high noise but similar mean was selected and called GY174. Thus, GY159 and GY174 theoretically contained only 6.25% of RM11-1a genome but had retained high-noise levels of the *P_MET17_-GFP* construct. The 55 spores used to validate QTL_3_ were obtained by crossing GY157 with BY4714. The strains used to demonstrate the effect of *ura3Δ0* on noise were GY244, GY246, GY333 and GY601. GY244 and GY246 were random spores from a cross between GY51 and BY4741. GY333 was obtained by transforming GY246 with a NotI restriction fragment from plasmid HO-hisG-URA3-hisG-poly-HO described in Voth et al. [42]. GY601 was obtained by amplifying the URA3 gene of BY4716 with primers 5′-AGGGAAGACAAGCAACGAAACGT-3′ and 5′-CCAGCCCATATCCAACTTCCAAT-3′ and transforming GY53 with this product. Strain GY321 was obtained by crossing GY172 (which was a spore from GY51×BY4710) with the *dst1* strain FY1671 kindly provided by F. Winston. We followed the kinetics of GY321 and GY51 growth in the physiological conditions of P_MET17_-GFP noise measurements and found identical growth rates (data not shown). For the complementation test of *dst1Δ*, the 4.6kb NotI fragment of plasmid pHO::DST1 was transformed in strain GY321 to give strain GY361. The corresponding negative control strain GY358 was obtained by transforming GY321 with the NotI fragment of the empty plasmid pHO-poly-KanMX4-HO. To test the effect of the *ura3-52* mutation on noise, strains GY51 and FY1679-18D were crossed and two random spores were selected: GY241 and GY243. To test the effect of *ura1Δ* and *ura2Δ* mutations, strain GY329 was obtained by amplifying the *ura1Δ::Kan^R^* mutation from the EUROSCARF strain *YKL216W* with primers 5′-CGGACGATAAACTTCGAAACAATTC-3′ and 5′-GGCACTTAACAATGTTTCGGAACTC-3′, and transforming strain GY51 with this amplicon; strain GY325 was obtained by amplifying the *ura2Δ::Kan^R^* mutation from the EUROSCARF strain *YJL130C* with primers 5′-GCGTATTTTAGTATCTGGGCGTGG-3′ and 5′-CGGACCTGATGTTACCTCCTTACTG-3′ and transforming strain GY51 with this amplicon. Similarly, strains GY602 to GY608 were constructed by amplifying the deletion mutation from the corresponding EUROSCARF strain with about 400bp flanking sequence, transforming GY51 with the amplicon, and checking proper integration by PCR with at least one primer designed outside the mutagenic fragment. We verified that Y9J_1 beard a functional *URA3* allele by amplifying it with primers 5′-AGGGAAGACAAGCAACGAAACGT-3′ and 5′-CCAGCCCATATCCAACTTCCAAT-3′ and transforming a *ura3Δ0* strain, which led to complementation of the ura-phenotype. We also checked that *ura3Δ0* and *dst1Δ* mutations did not change the fraction of cells in G1 by staining population of cells with propidium iodide as previously described[43], and analyzing distributions of DNA content by flow-cytometry (Figure S5).

### Flow Cytometry

4ml of YPD medium was inoculated with an isolated colony, and incubated overnight at 30°C with 220 rpm shaking. This starter culture was used to inoculate at OD_600_ = 0.1 4ml of autoclaved SD-MET medium [Yeast Nitrogen Base 6.7 g/L, Glucose 2%, Dropout Mix 2 g/L, adjusted to pH = 5.8 with NaOH] supplemented with 1 mM methionine (repressed condition). The Dropout Mix was a powder made of 2 g of uracil , 4 g of leucine, 1g of adenine, and 2 g of each of the following amino-acids: A, R, D, N, C, E, Q, G, H, I, K, F, P, S, T, W, Y, V. The culture was incubated at 30°C for exactly 3 hours with shaking, centrifuged at 1100×g for 5 minutes, and cells were resuspended in 4 ml of SD-MET medium supplemented with 50 µM methionine (moderate induction). Other methionine concentrations were tested in the experiments of Figure 4C–E (0, 20, 50, 100 and 200 µM). In the case of 6-AU treatments, the drug was added at this step to a final concentration of 100 µg/ml. In the case of increased uracil concentrations, uracil was added at both repressed and induced steps from a 2 mg/ml stock solution. The induced culture was incubated at 30°C for exactly 2 hours with shaking and a few micro-liters were analyzed on a FACSCAN (Beckton Dickinson) cytometer to record optical parameters of 15,000 living cells. The parameters were: Forward Scatter (FSC) on a linear scale, Side Scatter (SSC) on a linear scale, and GFP fluorescence (FL1) on a log scale. Raw data were read either directly from the original listmode data files using the RflowCyt package from Bioconductor (www.bioconductor.org), or from ASCII text files obtained after running MFI (Martz, Eric. 1992–2001. MFI: a flow cytometry list mode data analysis program optimized for batch processing under MS-DOS. http://www.umass.edu/microbio/mfi).

### Data Analysis

All computational analysis was done using the R statistical package (www.r-project.org). Because the distribution of FSC and SSC values differed slightly between the divergent genetic backgrounds, we did not gate the data but applied the following correction for cellular granularity and size: y_i_→ȳ+ε_i_, where y_i_ is the observed FL1value of the i^th^ cell and ε_i_ is the i^th^ residual of linear regression FL1 = ȳ+b*log(FSC)+c*log(SSC). The conclusions of the study remained if gating was applied instead of this correction (Figure S4). Noise was then defined as the coefficient of variation (standard deviation/mean ratio) of the corrected values.

### QTL Mapping

We searched for QTL by two complementary approaches: genome scanning and introgression. For genome scanning, the three noise values of each S288c×RM11-1a segregant were averaged and genetic linkage was searched at every marker position as follows: segregants were divided in two groups according to the marker genotype, noise difference between the two groups was tested using the Wilcoxon Mann-Whitney test. The genome-wide significance of the corresponding nominal *P*-values was determined by permuting the segregant indexes, re-scanning the genome and recording the smallest *P*-value obtained at each run. *P*<2.7×10^−5^ was reached in only 5 of the 500 permutation runs, thus defining the 1% genome-wide significance. For introgression, strains GY159 and GY174 were obtained by consecutive backcrosses with S288c derivatives, selecting spores with high-noise levels at each generation. GY159 and GY174 were then genotyped using oligonucleotide microarrays: their genomic DNA was extracted, digested, labeled and hybridized to YGS98 Affymetrix® Yeast Genome microarrays as described previously[44]. The genotype of each strain was obtained at 3015 marker positions by adding the corresponding raw .CEL data file to the dataset of Yvert *et al.* 2003[45] and by applying the same algorithm as previously described in Brem et al. 2002[2]. We then screened the markers for those harboring the RM11-1a genotype in the two introgressed strains (GY159 and GY174) as well as in the S288c×RM11-1a segregant displaying the highest noise level (GY157). A total of 230 markers were selected this way, 32 of them being clustered at one locus on chromosome V (Figure 2B). To determine if the other 198 markers, which were scattered across the genome, truly reflected RM11-1a genotypes, we directly assessed them by PCR and sequencing or RFLP. We found that most of these markers were in fact of the S288c genotype in at least one of the two introgressed strains and we did not consider them further. The locus on chromosome V was then validated as a QTL of *P_MET17_-GFP* noise by analyzing an independent cross as described in text.

### Estimation of *ura3Δ0* Contribution to Noise Decoupled from Mean Effects

Because noise scaled with mean expression, we used various induction levels of the reporter construct by varying the concentration of the repressor (methionine). The data presented on Figure 4C was then treated as follows: a linear model was fitted to S288c values (red), and noise values from the two other strains (blue) were corrected by subtracting the expected noise value from the model. Corrected noise values were then averaged for each strain, estimating at 3.5% the difference between S288c and RM11-1a, and at 2.2% the difference between S288c and the *URA3*-rescued RM11-1a strain (note that here the phenotype itself is measured as a percentage since it is a coefficient of variation). The *ura3Δ0* mutation therefore contributed to (3.5–2.2)/3.5 = 37% of the total difference between the parental backgrounds.

## Supporting Information

Figure S1 Genetic variation of noise when integrating the reporter construct at the LYS2 locus. Strains GY122 and GY125 carried the PMET17-GFP construct at the LYS2 locus instead of the HIS3 locus and were derived from S288c and RM11-1a, respectively. Results were strictly comparable to the ones obtained from HIS3:PMET17-GFP strains, with a similar difference in noise between the two backgrounds and no particular variation of mean expression level.
(0.27 MB EPS)

Figure S2 High noise levels in ura3 spores from CEN.PK×FL200. Tetrads were dissected from a GY43xGY44 hybrid strain, and were analyzed by flow cytometry for PMET17-GFP noise levels. Spores that inherited the ura3 mutation from GY44 (triangles) showed higher noise than their siblings (crosses). Dashed and continuous lines represent linear fit to Ura+ and Ura− data points, respectively.
(0.27 MB EPS)

Figure S3 A) Comparison of PMET17-GFP noise and mean expression levels between strains GY241 and GY243 that were isogenic except for the specified ura3 genotypes. The ura3-52 mutation is associated to higher noise (P = 0.04) without affecting mean expression. B) Strain GY53 was analyzed by flow cytometry in media containing increasing concentration of uracil. C) Comparison of PMET17-GFP noise and mean expression levels between strains GY51, GY329 and GY325 that were isogenic except for the specified ura1 and ura2 genotypes.
(0.30 MB EPS)

Figure S4 Noise differences observed from cells of similar size. A) FSC/SSC scatter plot of two representative experiments of strain GY51 (red) and GY53 (blue). Two gates were visually chosen and cells falling in each gate were extracted from the dataset (which corresponded to about 200 cells for each experiments). B) same representation as in A) but from two representative experiments of strains GY246 (red) and GY244 (blue). C) same representation as in A) and B) but from two representative experiments of strains GY321 (red) and GY51 (blue). D–E) Boxplots displaying PMET17-GFP noise estimates (standard deviation/mean of raw fluorescence values) from the gated cells selected in A). F–G) Boxplots displaying PMET17-GFP noise estimates (standard deviation/mean of raw fluorescence values) from the gated cells selected in B). H–I) Boxplots displaying PMET17-GFP noise estimates (standard deviation/mean of raw fluorescence values) from the gated cells selected in C). The genetic variation of noise is visible from all gated subdatasets.
(14.00 MB EPS)

Figure S5 ura3 and dst1 mutations do not perturb cell-cycle progression distributions. Cells were cultured as for PMET17-GFP noise measurements and were fixed and stained with propidium iodide (PI) to quantify their DNA content (FL2-A channel). The distribution of PI fluorescence is shown for strains GY246 (A), GY244 (B), GY321 (D) and GY51 (E). Bottom panels show quantile-quantile plots (red) comparing the two above distributions. Dashed diagonal line represents identity. C) Comparison of ura3�?0 strain GY246 to URA3 wild-type strain GY244. D) Comparison of dst1�? strain GY321 to DST1 wild-type strain GY51. The distributions do not differ significantly within the G1-S-G2/M window (framed by dashed vertical lines across the panels).
(3.06 MB EPS)

Table S1 Strains used in this study.
(0.13 MB DOC)

## References

[1] Damerval C, Maurice A, Josse JM, de Vienne D (1994). Quantitative trait loci underlying gene product variation: a novel perspective for analyzing regulation of genome expression.. Genetics.

[2] Brem RB, Yvert G, Clinton R, Kruglyak L (2002). Genetic dissection of transcriptional regulation in budding yeast.. Science.

[3] Klose J, Nock C, Herrmann M, Stuhler K, Marcus K (2002). Genetic analysis of the mouse brain proteome.. Nat Genet.

[4] Li Y, Alvarez OA, Gutteling EW, Tijsterman M, Fu J (2006). Mapping determinants of gene expression plasticity by genetical genomics in C. elegans.. PLoS Genet.

[5] DeCook R, Lall S, Nettleton D, Howell SH (2006). Genetic regulation of gene expression during shoot development in Arabidopsis.. Genetics.

[6] Schadt EE, Monks SA, Drake TA, Lusis AJ, Che N (2003). Genetics of gene expression surveyed in maize, mouse and man.. Nature.

[7] Cheung VG, Spielman RS, Ewens KG, Weber TM, Morley M (2005). Mapping determinants of human gene expression by regional and genome-wide association.. Nature.

[8] Schadt EE, Lamb J, Yang X, Zhu J, Edwards S (2005). An integrative genomics approach to infer causal associations between gene expression and disease.. Nat Genet.

[9] Kulp DC, Jagalur M (2006). Causal inference of regulator-target pairs by gene mapping of expression phenotypes.. BMC Genomics.

[10] Lee SI, Pe'er D, Dudley AM, Church GM, Koller D (2006). Identifying regulatory mechanisms using individual variation reveals key role for chromatin modification.. Proc Natl Acad Sci U S A.

[11] Kwan T, Benovoy D, Dias C, Gurd S, Provencher C (2008). Genome-wide analysis of transcript isoform variation in humans.. Nat Genet..

[12] Spudich JL, Koshland DE (1976). Non-genetic individuality: chance in the single cell.. Nature.

[13] Elowitz MB, Levine AJ, Siggia ED, Swain PS (2002). Stochastic gene expression in a single cell.. Science.

[14] Blake WJ, M KA, Cantor CR, Collins JJ (2003). Noise in eukaryotic gene expression.. Nature.

[15] Raj A, Peskin CS, Tranchina D, Vargas DY, Tyagi S (2006). Stochastic mRNA synthesis in mammalian cells.. PLoS Biol.

[16] Suel GM, Garcia-Ojalvo J, Liberman LM, Elowitz MB (2006). An excitable gene regulatory circuit induces transient cellular differentiation.. Nature.

[17] Acar M, Becskei A, van Oudenaarden A (2005). Enhancement of cellular memory by reducing stochastic transitions.. Nature.

[18] Sigal A, Milo R, Cohen A, Geva-Zatorsky N, Klein Y (2006). Variability and memory of protein levels in human cells.. Nature.

[19] Kaufmann BB, Yang Q, Mettetal JT, van Oudenaarden A (2007). Heritable Stochastic Switching Revealed by Single-Cell Genealogy.. PLoS Biol.

[20] Arias AM, Hayward P (2006). Filtering transcriptional noise during development: concepts and mechanisms.. Nat Rev Genet.

[21] Bahar R, Hartmann CH, Rodriguez KA, Denny AD, Busuttil RA (2006). Increased cell-to-cell variation in gene expression in ageing mouse heart.. Nature.

[22] Raser JM, O'Shea EK (2004). Control of Stochasticity in Eukaryotic Gene Expression.. Science.

[23] Blake WJ, Balazsi G, Kohanski MA, Isaacs FJ, Murphy KF (2006). Phenotypic consequences of promoter-mediated transcriptional noise.. Mol Cell.

[24] Colman-Lerner A, Gordon A, Serra E, Chin T, Resnekov O (2005). Regulated cell-to-cell variation in a cell-fate decision system.. Nature.

[25] Bean JM, Siggia ED, Cross FR (2006). Coherence and timing of cell cycle start examined at single-cell resolution.. Mol Cell.

[26] Guido NJ, Lee P, Wang X, Elston TC, Collins JJ (2007). A pathway and genetic factors contributing to elevated gene expression noise in stationary phase.. Biophys J.

[27] Newman JR, Ghaemmaghami S, Ihmels J, Breslow DK, Noble M (2006). Single-cell proteomic analysis of S. cerevisiae reveals the architecture of biological noise.. Nature.

[28] Bar-Even A, Paulsson J, Maheshri N, Carmi M, O'Shea E (2006). Noise in protein expression scales with natural protein abundance.. Nat Genet.

[29] Brem RB, Kruglyak L (2005). The landscape of genetic complexity across 5,700 gene expression traits in yeast.. Proc Natl Acad Sci U S A.

[30] Li J, Burmeister M (2005). Genetical genomics: combining genetics with gene expression analysis.. Hum Mol Genet.

[31] Rose M, Winston F (1984). Identification of a Ty insertion within the coding sequence of the S. cerevisiae URA3 gene.. Mol Gen Genet.

[32] Louis E, Durbin R (2007). http://www.sanger.ac.uk/Teams/Team71/durbin/sgrp/.

[33] Mason PB, Struhl K (2005). Distinction and relationship between elongation rate and processivity of RNA polymerase II in vivo.. Mol Cell.

[34] Qiu H, Hu C, Wong CM, Hinnebusch AG (2006). The Spt4p subunit of yeast DSIF stimulates association of the Paf1 complex with elongating RNA polymerase II.. Mol Cell Biol.

[35] Joshi AA, Struhl K (2005). Eaf3 chromodomain interaction with methylated H3-K36 links histone deacetylation to Pol II elongation.. Mol Cell.

[36] Voliotis M, Cohen N, Molina-Paris C, Liverpool T (2007). Fluctuations, pauses and backtracking in DNA transcription.. Biophys J.

[37] Wagner A (2005). Robustness and Evolvability in Living Systems..

[38] Zlotogora J (2003). Penetrance and expressivity in the molecular age.. Genet Med.

[39] Narod SA, Foulkes WD (2004). BRCA1 and BRCA2: 1994 and beyond.. Nat Rev Cancer.

[40] Cormack BP, Bertram G, Egerton M, Gow NA, Falkow S (1997). Yeast-enhanced green fluorescent protein (yEGFP)a reporter of gene expression in Candida albicans.. Microbiology.

[41] Brachmann CB, Davies A, Cost GJ, Caputo E, Li J (1998). Designer deletion strains derived from Saccharomyces cerevisiae S288C: a useful set of strains and plasmids for PCR-mediated gene disruption and other applications.. Yeast.

[42] Voth WP, Richards JD, Shaw JM, Stillman DJ (2001). Yeast vectors for integration at the HO locus.. Nucleic Acids Res.

[43] Nash R, Tokiwa G, Anand S, Erickson K, Futcher AB (1988). The WHI1+ gene of Saccharomyces cerevisiae tethers cell division to cell size and is a cyclin homolog.. Embo J.

[44] Winzeler EA, Richards DR, Conway AR, Goldstein AL, Kalman S (1998). Direct allelic variation scanning of the yeast genome.. Science.

[45] Yvert G, Brem RB, Whittle J, Akey JM, Foss E (2003). Trans-acting regulatory variation in Saccharomyces cerevisiae and the role of transcription factors.. Nat Genet.

