# Supplementary material for: Cell-to-Cell Stochastic Variation in Gene Expression Is a Complex Genetic Trait
Source: PLoS Genet. 2008 Apr 11;4(4):e1000049. doi: 10.1371/journal.pgen.1000049 (PMC2289839; doi:10.1371/journal.pgen.1000049)
Supplement: Table S1 — Strains used in this study. (0.13 MB DOC) [file pgen.1000049.s006.doc]

**Table S1. Strains used in this study.**

| Name | General Background | Specific genotypes | Source |
| --- | --- | --- | --- |
| BY4709 | S288c | *MATalpha ura3∆0* | *Brachmann et al. [41]* |
| BY4712 | S288c | *MATa leu2∆0* | *Brachmann et al. [41]* |
| BY4713 | S288c | *MATalpha leu2∆0* | *Brachmann et al. [41]* |
| BY4714 | S288c | *MATa his3∆200* | *Brachmann et al. [41]* |
| BY4715 | S288c | *MATa lys2∆0* | *Brachmann et al. [41]* |
| BY4716 | S288c | *MATalpha lys2∆0* | *Brachmann et al. [41]* |
| BY4719 | S288c | *MATa trp1∆63 ura3∆0* | *Brachmann et al. [41]* |
| BY4741 | S288c | *MATa his3∆1 leu2∆0 met15∆0 ura3∆0* | *Brachmann et al. [41]* |
| BY4742 | S288c | *MATalpha his3∆1 leu2∆0 lys2∆0 ura3∆0* | *Brachmann et al. [41]* |
| CEN.PK113-5D | CEN.PK | *MATa ura3-52* | *P. Hieter* |
| FL200 | FL200 | *MATalpha* | *Casaregola et al. [46]* |
| FY1671 | S288c | *MATalpha leu2∆1 lys2-128d ura3-52 his4-912d dst1∆::hisG* | *F. Winston* |
| FY1679-18D | S288c | *MATa his3∆200 ura3-52* | *B. Dujon* |
| FY67 | S288c | *MATa trp1∆63* | *F. Winston* |
| FYC20-2A | S288c | *MATa his3∆200 lys2∆202* | *B. Dujon* |
| GY122 | S288c | *MATalpha ura3∆0 lys2∆:: (natMX + Pmet17-yEGFP3)* | *this study* |
| GY125 | RM11-1a | *MATa leu2∆0 ura3∆0 ho::KanMX amn1-A1103T lys2∆:: (natMX + Pmet17-yEGFP3)* | *this study* |
| GY157 | S288c(50%) x RM11-1a (50%) | *MATalpha lys2∆0 ura3∆0 HIS3:(natMX + Pmet17-yEGFP3):HIS3* | *this study* |
| GY159 | S288c (94%), RM11-1a (6%) | *MATa leu2∆0 lys2∆202 trp1∆63 ura3∆0 HIS3:(natMX + Pmet17-yEGFP3):HIS3* | *this study* |
| GY172 | S288c | *MATa trp1∆63 HIS3:(natMX + Pmet17-yEGFP3)* | *this study* |
| GY174 | S288c (94%), RM11-1a (6%) | *MATa ura3∆0 HIS3:(natMX + Pmet17-yEGFP3):HIS3* | *this study* |
| GY241 | S288c | *MATalpha lys2∆0 ura3-52 HIS3:Pmet17-yEGFP3-NatMX:HIS3* | *this study* |
| GY243 | S288c | *MATalpha lys2∆0 URA3 HIS3:Pmet17-yEGFP3-NatMX:HIS3* | *this study* |
| GY244 | S288c | *MATa leu2∆0 URA3 HIS3:(natMX + Pmet17-yEGFP3):HIS3* | *this study* |
| GY246 | S288c | *MATa leu2∆0 ura3∆0 HIS3:(natMX + Pmet17-yEGFP3):HIS3* | *this study* |
| GY321 | S288c | *MATa lys2-128d dst1∆::hisG HIS3:(natMX + Pmet17-yEGFP3)* | *this study* |
| GY325 | S288c | *MATalpha lys2∆0 HIS3:(natMX + Pmet17-yEGFP3) ura2∆::KanMX* | *this study* |
| GY329 | S288c | *MATalpha lys2∆0 HIS3:(natMX + Pmet17-yEGFP3) ura1∆::KanMX* | *this study* |
| GY333 | S288c | *MATa leu2∆0 ura3∆0 HIS3:(natMX + Pmet17-yEGFP3):HIS3 ho∆::hisG-URA3-hisG* | *this study* |
| GY356 | RM11-1a | *MATa leu2∆0 ura3∆0 ho::KanMX amn1-A1103T*  *HIS3:(natMX + Pmet17-yEGFP3):HIS3* | *this study* |
| GY358 | S288c | *MATa lys2-128d dst1∆::hisG HIS3:(natMX + Pmet17-yEGFP3) ho::KanMX4* | *this study* |
| GY361 | S288c | *MATa lys2-128d dst1∆::hisG HIS3:(natMX + Pmet17-yEGFP3) ho::(DST1+KanMX4)* | *this study* |
| GY43 | FL200 | *MATalpha HIS3: (natMX + Pmet17-yEGFP3)* | *this study* |
| GY44 | CEN.PK | *MATa ura3-52 HIS3:(natMX + Pmet17-yEGFP3)* | *this study* |
| GY445 | Y9J_1 | *MATa/MATalpha HIS3/HIS3:(natMX + Pmet17-yEGFP3)* | *this study* |
| GY51 | S288c | *MATalpha lys2∆0 HIS3:(natMX + Pmet17-yEGFP3)* | *this study* |
| GY53 | RM11-1a | *MATa leu2∆0 ura3∆0 amn1∆::HYG ho::KAN HIS3:(natMX + Pmet17-yEGFP3)* | *this study* |
| GY601 | RM11-1a | *MATa leu2∆0 URA3 amn1∆::HYG ho::KAN HIS3:(natMX + Pmet17-yEGFP3)* | *this study* |
| GY602 | S288c | *MATalpha lys2∆0 HIS3:(natMX + Pmet25-yEGFP3) trp1∆::KanMX4* | *this study* |
| GY603 | S288c | *MATalpha lys2∆0 HIS3:(natMX + Pmet25-yEGFP3) eaf3∆::KanMX4* | *this study* |
| GY604 | S288c | *MATalpha lys2∆0 HIS3:(natMX + Pmet25-yEGFP3) spt4∆::KanMX4* | *this study* |
| GY605 | S288c | *MATalpha lys2∆0 HIS3:(natMX + Pmet25-yEGFP3) leo1∆::KanMX4* | *this study* |
| GY606 | S288c | *MATalpha lys2∆0 HIS3:(natMX + Pmet25-yEGFP3) set2∆::KanMX4* | *this study* |
| GY607 | S288c | *MATalpha lys2∆0 HIS3:(natMX + Pmet25-yEGFP3) ccr4∆::KanMX4* | *this study* |
| GY608 | S288c | *MATalpha lys2∆0 HIS3:(natMX + Pmet25-yEGFP3) cdc73∆::KanMX4* | *this study* |
| YEF1685^(i)^ | RM11-1a | *MATa leu2∆0 ura3∆0 ho::KANr amn1::HYG* | *E. Foss* |
| YEF1946^(i)^ | RM11-1a | *MATa leu2∆0 ura3∆0 ho::KanMX amn1-A1103T* | *E. Foss* |

(i): Because strains YEF1946 and YEF1685 were both used as derivatives of RM11-1a in which clumpyness was suppressed [45], we compared strains GY53 and GY356 and found that *amn1* genotype did not influence *P_MET17_-GFP* noise nor mean levels (data not shown).

References:

41. Brachmann CB, Davies A, Cost GJ, Caputo E, Li J, et al. (1998) Designer deletion strains derived from Saccharomyces cerevisiae S288C: a useful set of strains and plasmids for PCR-mediated gene disruption and other applications. Yeast 14: 115-132.

45. Yvert G, Brem RB, Whittle J, Akey JM, Foss E, et al. (2003) Trans-acting regulatory variation in Saccharomyces cerevisiae and the role of transcription factors. Nat Genet 35: 57-64.

46. Casaregola S, Nguyen HV, Lepingle A, Brignon P, Gendre F, et al. (1998) A family of laboratory strains of Saccharomyces cerevisiae carry rearrangements involving chromosomes I and III. Yeast 14: 551-564.
